# Psychosocial predictors of physical activity and health-related quality of life among Shanghai working adults

**DOI:** 10.1186/s12955-019-1145-6

**Published:** 2019-04-25

**Authors:** Yi Xiao, Hongying Wang, Tao Zhang, Xiaoling Ren

**Affiliations:** 1 Shanghai University of Sport, NO. 399, Changhai Road, Yangpu District, Shanghai, 200438 People’s Republic of China; 2 University of North Texas, Denton, USA

**Keywords:** Stress, Social support, Self-efficacy, Physical activity, Shanghai community

## Abstract

**Background:**

Participating in regular physical activity contributes to significant improvements of quality of life (QOL) in adults. Understanding psychosocial factors that influence physical activity and QOL in working adults may have important implications for future interventions aimed at improving their health. The major purpose of this study was to investigate the psychosocial predictors of physical activity and QOL among Shanghai working adults.

**Methods:**

Participants were 238 working adults (*M*_age_ = 51.6 ± 5.6) living in Shanghai communities, China. They completed previously validated questionnaires assessing their perceptions of stress, social support from friends, self-efficacy, physical activity, and QOL. Pearson correlations were computed to assess the associations among physical activity, QOL, and psychosocial variables. Path analysis was used to test the predictive strengths of psychosocial factors on physical activity and QOL among Shanghai working adults.

**Results:**

The results indicated that stress had directly negative relationships on self-efficacy and QOL. Social support had directly positive relationships on self-efficacy, physical activity, and QOL. Physical activity had directly positive relationship on QOL. Self-efficacy and physical activity mediated the influences of stress and social support on QOL.

**Conclusions:**

Stress and social support from friends were two important sources of self-efficacy, all of which facilitated more physical activity participation. Lower stress, higher social support, and more physical activity may directly increase QOL among Shanghai working adults. The mediating roles of self-efficacy and physical activity should be taken into account in managing stress and social support in order to promote QOL among Shanghai working adults.

## Introduction

With the rapid development of economy, Shanghai has been experiencing great changes during the last few decades as one of the most industrialized cities in China. As a result, the living conditions have been improved greatly, and many workers are getting richer than before [1]. On the other hand, workers living in Shanghai are also encountering the negative effects of this rapid development. More and more workers from other cities swarmed into Shanghai to look for jobs and live, which makes the city more crowded, and the working environment more competitive than ever before [1]. The working adults in Shanghai are experiencing growing levels of stress, decreased levels of physical activity, and reduced levels of QOL [2].

It is well-documented that physical inactivity is principal major risk factor for global mortality and is associated with 3.2 million deaths per year [3]. Due to declines in physical activity, the health benefits of regular physical activity are beginning to be increasingly recognized by the medical and health community [4]. Previous studies have reported positive relationships between physical activity and various health indicators [5, 6]. Participating in regular physical activity can not only reduce the morbidity and mortality of chronic diseases, but also improve an individual’s quality of life [7, 8]. Physical activity can contribute to alleviating negative outcomes, which in turn could significantly improve the levels of QOL. Engaging in regular physical activity is effectively alleviate stress, improves self-efficacy, and results in higher levels of QOL [9, 10].

Factors that may influence the initiation of and adherence to physical activity include age, gender, physical limitations, socioeconomic factors, psychosocial factors such as self-efficacy and social support, and environmental factors such as safety and equipment accessibility [11]. Generally, the factors that influence individuals’ physical activity can be categorized into three types: intra-personal (e.g., psychological conditions and self-efficacy), inter-personal (e.g., social support from friends and family), and environmental constraints (e.g., lack of access to physical activity facilities) [4]. In the physical activity literature, social support and self-efficacy were important predictors of physical activity [4, 12]. Lack of social support and low self-efficacy are two important barriers to regular physical activity and QOL [4]. Based on a path analysis, Brannagan [13] examined the strength and directional relationship among exercise self-efficacy, stress and physical activity. They found that the relationships between stress, perceived exertion and physical activity are mediated by exercise self-efficacy. Based on social cognitive theory, Emily and Mailey investigated the physical activity intervention effects on perceived stress in working mother, they found that increases in physical activity were sustained and shown to be mediated by changes in self-efficacy and self-regulation [10]. Self-efficacy also acted as a mediator in the relationship between social support and health promoting behaviors such as physical activity. Silva and Lott [14] investigated the direct and indirect effects of social support on youth physical activity behavior, they found that peer social supports had direct effect on physical activity and self-efficacy mediated the influence on physical activity.

Above mentioned studies suggest that physical activity and QOL are both associated with many psychosocial factors such as working conditions, personality traits, social environment, and cognitive perceptions. The relationships among these factors are direct and/or indirect. However, many of these previous studies were conducted in the context of the western Judeo-Christian culture which places a high value on independence and self-realization. In Asia, the traditional Confucian teaching emphasizes interdependence and group harmony [1]. Thus, the findings of studies conducted in western countries may not be appropriate to a non-Western culture. The main purpose of the present study, therefore, was to investigate the psychosocial predictors of physical activity and QOL in Shanghai working adults, and examine the direct and indirect influences of these predictors on physical activity and QOL. It is anticipated that the findings can help provide significant insights into developing positive coping strategies, preventing stress, improving social support and self-efficacy perceptions, facilitating physical activity and finally improving their QOL. Understanding the psychosocial factors that influence physical activity and QOL in working adults could have particularly important implications for future interventions aimed at developing coping strategies to prevent stress, fostering good social relationship, improving their exercise self-efficacy, facilitating their physical activity, and finally improve their levels of QOL [15], especially in a non-Western culture city such as Shanghai, the financial center in China.

Three hypotheses were put forward in this study.

H1: Stress had directly negative relationships on self-efficacy and QOL.

H2: Social support had directly positive relationships on self-efficacy, physical activity, and QOL.

H3: Self-efficacy and physical activity mediated the influences of stress and social support on QOL.

## Method

### Settings and participants

Approximately 221 participants were needed for this study to reach a power of 85% at an alpha level of 0.05 (two tailed) based on a statistical power analysis to detect a correlation of r = 0.20 [16]. Thus, participants were 238 (99 males, 139 females; *M*_age_ = 51.6 ± 5.6) working adults who were randomly recruited from eight Shanghai communities using the stratified cluster sampling method. The sampling inclusion criteria were: aged 30 to 65 years, Shanghai residents, and having the ability to complete the health outcomes questionnaires in Chinese with accepted reliabilities and validities. 423 working adults were invited to attend the study. Informed assent forms were distributed to all 423 participants prior to data collection. A total of 321 working adults agreed to participate. Participants spent approximately 30 min completing the questionnaires. The questionnaires were collected immediately upon completion. A total of 296 participants out of the 321 working adults voluntarily returned the survey, which yielded a return rate of 92.21%. Of the 296 participants, fifty-eight participants were excluded because of missing and incomplete answers. The final analytic sample consisted of 238 participants. This study obtained the approval of ethics committee in Shanghai university of Sport. All participants signed consent forms before they joined this study.

## Measures

### Demographic variable

To characterize the participants in this study, personal information including gender, age and so on was obtained through face to face interview.

### Stress

Stress refers to the internal perceived emotions and cognition, which is usually considered to be the response to a stimulus. Stress occurs when an individual perceives that environmental demands tax or exceed one’s adaptive capacity [17]. Although certain levels of stress can inspire people to achieve great success, excessive stress may lead to depression, burnout, and reduced QOL, which in turn induces absenteeism and increased health expenses [18]. Self-efficacy and stress are closely related concepts. A strong negative relationship between self-efficacy and perceived stress was postulated [19, 20]. Research indicated that reducing stress tends to have a positive influence on increasing self-efficacy [20]. While higher working stress can cause the poorer physical health and work performance and then cause the midwife’s self-efficacy to decrease [21]. Stress has been shown as an important predictor of self-efficacy. Physiological arousal states related to stress provide information affecting self-efficacy judgments [22]. In addition, increased stress in the psychological, physical, and service areas leads to reduced self-efficacy [23].

Perceived stress was measured by the 10-item Perceived Stress Scale (PSS-10), which consists of six negative and four positive items. Participants are asked to respond to each question on a 5-point Likert scale ranging from 0 (never) to 4 (very often), indicating how often they have felt or thought a certain way within the past month. The PSS-10 scores are obtained by reversing the scores on the four positively stated items, and then summed across all 10 items. The higher total scores indicate greater perceived stress [24].

### Exercise social support

Social support can be defined as the contact, assistance, and/or information one receives through formal and informal contacts with individuals and groups, which involves the provisions of aid and assistance exchanged through social relationships and interpersonal transactions [25]. Social support has a meaningful and positive influence on overall QOL. The stronger the social support an individual gets at home and work, the greater perceived QOL he/she has [1]. Poor social support is significantly related to lower QOL. Studies have suggested that social support may have a mitigating effect on distress [26]. Otherwise, social support, which increases self-efficacy, has been shown to be helpful for those who facing stressful situations [27]. Mary’s study indicated that social support acts as a social buffer against stress, it seems to strengthen the physical, mental, social and psychological well-being of inpatient caregivers [28]. In addition, those who had greater social support from friends and family members participated in higher levels of physical activity. Lack of social support and low self-efficacy are important barriers to regular physical activity [29].

The Exercise Social Support survey [30] was used to measure social support from friends, which has 13 items rated on a 5-point Likert scale ranging from 1 (none) to 5 (very often). Item scores are coded and summed up to get the total score of exercise social support from friends, with a higher averaged total score indicating greater social support from friends.

### Exercise self-efficacy

Self-efficacy is a self-estimation of one’s ability to successfully execute necessary actions to achieve desired outcomes. Self-efficacy was significantly and positively related to an individual’s initiation, participation, and self-regulation in physical activity. It played a critical role in changing old lifestyles and initiating new physical activity behaviors, and would determine whether new physical activity behaviors will be motivated, how long individuals will persist when facing aversive experiences, and how much effort they will put in physical activities [31].With regards to physical activity, individuals with higher exercise-specific self-efficacy are likely to participate in more exercise, expend more effort, persist longer, show greater interest in exercise, and achieve at higher levels of physical activity than those who doubt their capabilities of exercise performance in facing difficulties and obstacles [32]. Moreover, self-efficacy has been examined as a mediator between social support and physical activity [33].

The exercise self-efficacy measure was designed to tap participants’ self-efficacy with respect to continued exercise participation (at least three times per week for 40 min at moderate intensity) over incremental week periods for 8 weeks. This measure has also been shown to be predictive of exercise behavior [33] and internally consistent. Participants indicated their degree of confidence for eight items on a scale ranging from 0% (no confidence at all) to 100% (completely confident). It has good internal consistency reliability and reliability.

### Physical activity

The short self-administered version of the International Physical Activity Questionnaire IPAQ (IPAQ-SF) was used to measure physical activity. The IPAQ-SF is a 7-item scale, assessing the amount of minutes spent in walking, in vigorous and moderate intensity activity, and in sedentary activity during the last 7 days. For all categories, participants have to define on how many days and how many minutes they spent at a specific activity category. For all categories, the amount of Metabolic Equivalents (METs)-minutes is calculated by multiplying the amount of minutes with 8 (vigorous), 4 (moderate), 3.3 (walking), or 1.3 (sitting), respectively [34]. Besides these four subscores, a total score is calculated by summing the METs-minutes of the first 3 categories together. To improve the normality distribution for energy expenditure, a logarithmic transformation was used due to the non-normal distribution of energy expenditure of participants’ physical activity. The IPAQ-SF has good test-retest reliability and moderate criterion validity in healthy adults [35].

### Quality of life

Quality of life (QOL) refers to how individuals subjectively perceive the negative and positive aspects of their lives and includes both physical and mental factors that collectively affect one’s perception of the overall satisfaction with his/her life [36]. It is a multidimensional concept that incorporates physical and psychological well-being, social relationship, lifestyle factors, and people’s expectation for their life [37]. Demographic variables such as age and gender have not been found to be related to QOL [38]. But psychological and physical health status, personality traits, cognitive ability, and socio-demographic factors may be psychosocial and health determinants [15]. Meanwhile, educational level, number of chronic diseases, physical performance, and number of caregivers had a significant impact on the four domain scores of the QOL [39]. Exploration indicate that continued stress can lead to job burnout, physical and psychological illness, and finally decreased QOL [18].

QOL was measured using the Chinese version of Quality of Life Scale-Brief [40], which was developed as a short form of the WHOQOL-100 and translated into Chinese so that it would be suitable for Chinese elderly population. It is a self-reported questionnaire containing 26 questions, each representing one facet of the WHOQOL-100, as well as one facet on overall quality of life, and one on general health. Each item is rated on a five-point Likert scale (1 = very dissatisfied to 5 = very satisfied). It produces scores for four domains related to QOL: physical (physical health and functional status), psychological (psychological well-being), social relationships (personal relationships and social support) and environment. Item scores for each domain are coded and summed up to get the total QOL score, with a higher total score indicating better quality of life.

## Procedure

The participants who signed the consent form were given a full explanation about the study purpose, the potential benefits/risks, the confidentiality, and withdrawal rights. After that, they were directed to complete the questionnaires of stress, social support, exercise self-efficacy, physical activity, and QOL. To minimize participants’ propensity to offer socially desirable responses, they were encouraged to complete the surveys as honestly and completely as possible. They were also confirmed that their responses would be used only for research.

## Path Aznalysis

To evaluate the fit of the model to the data, various indices of fit were examined. Specifically, the chi-square statistic (χ^2^) tests whether there is a statistically significant difference between the model and sample data and degrees of freedom (*df*) for each model estimated. Further, values less than .08 obtained from the RMSEA suggest a well-fit model, whereas values exceeding .10 are typically undesirable. Finally, possible values for CFI, RFI, NFI, and GFI fit indices range between 0 and 1. CFI, RFI, NFI, and GFI values greater than .90 indicate a good fit of the model to the data, and values greater than .95 are typically considered an excellent fit [41].

## Data analysis

**Fig. 1:**
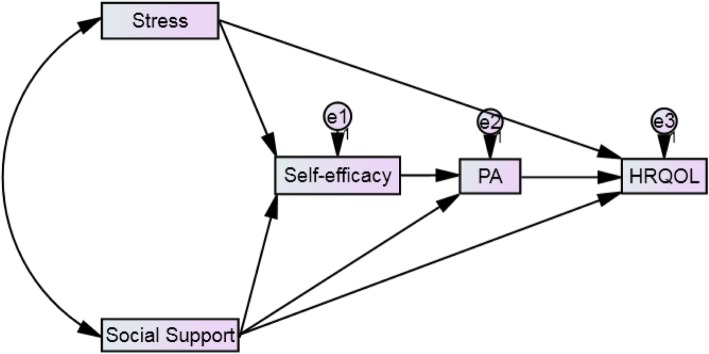
Hypothesized Model of the Variables (N = 238). *Note.* Solid lines represent significant standardized parameter estimates. Squares represent observed variables. PA = physical activity; HRQOL = health-related quality of life

Data were analyzed by the Statistical Product and Service Solutions (SPSS 22.0, SPSS Inc.). Descriptive statistics and internal consistency estimates (Cronbach’s alpha) were conducted on all study variables. Pearson product-moment correlation was computed to assess the strengths of association between physical activity, QOL, and three psychosocial predictors (stress, social support, and self-efficacy). Using Analysis of Moment Structures (AMOS) version 22.0, all psychosocial variables from correlation matrices were analyzed to examine the hypothesized model described in Fig. 1 using path analysis. According to the suggestion of Bentler [42], various indices for model data fit were examined to access the model’s goodness-of-fit to the data. These indices included the chi-square statistic (χ^2^), Root Mean Square Error of Approximation (RMSEA), Comparative Fit Index (CFI), Relative Fit Index (RFI), Bentler-Bonett Non-normed Fit Index (NFI), and Goodness of Fit Index (GFI).

## Results

### T-test analysis and scale reliability

According to the age division of Australian Bureau of Statistics, age ≤ 44 years old, and age between 45 and 64 years old were divided into middle-aged, and older adults group respectively [43]. No significant difference was found between different age groups for BMI, react time and VO_2_Max (*p* = 0.400, *p* = 0.102, *p* = 0.822, respectively, Table 1). In addition, there was no significant difference between different age groups in terms of stress, social support, self-efficacy, physical activity and QOL (all *p* > 0.05, Table 1).

**Table 1:** One-way analysis of variance for different age groups (T-Test) (*N* = 238)

Variables	Middle-aged(*N* = 37)	Older adults(*N* = 201)	*p*-value
	M ± SD	M ± SD	
Age	41.92 ± 1.498	53.31 ± 4.027	0.000
Height (cm)	162.484 ± 8.391	164.353 ± 7.137	0.064
Weight (kg)	65.862 ± 11.55	66.348 ± 10.493	0.522
BMI	24.857 ± 3.19	24.48 ± 2.954	0.400
React time (sec)	0.444 ± 0.059	0.467 ± 0.068	0.102
VO_2_Max	24.973 ± 3.775	23.622 ± 3.874	0.822
Stress	20.35 ± 6.201	21.06 ± 5.348	0.172
Social support	5.331 ± 1.279	5.417 ± 1.312	0.874
Self-efficacy	74.757 ± 29.967	79.02 ± 26.719	0.162
Physical activity	2.731 ± 0.888	2.856 ± 0.847	0.703
QOL	93.324 ± 10.32	91.114 ± 11.011	0.429

Alpha coefficients for each measure are presented in Table 2. The Cronbach’s alpha coefficients of stress, social support, self-efficacy, and QOL scales were .79, .90, .99, and .85, respectively. As shown, self-report measures demonstrated acceptable levels of reliability, exceeding Nunnally’s [44] criterion of .70. In addition, the descriptive statistics indicated that the self-reported variables were above the midpoint.

**Table 2:** Descriptive statistics, internal consistency, and correlations among variables (N = 238)

Subscale	Range	1	2	3	4	5
1. Stress	0–40	(.79)				
2. Social support	1–5	- .18*	(.90)			
3. Self-efficacy	0–100	- .21**	.20**	(.99)		
4. Physical activity	0–4	- .02	.20**	.20**	(NA)	
5. HRQOL	26–130	- .48**	.31**	.19**	.17*	(.85)
*M*		20.95	2.61	78.36	2.84	91.46
*SD*		5.48	.77	27.23	.85	10.91

*Note.* Cronbach’s alpha coefficients are provided along the diagonal; HRQOL = health-related quality of life; *M* mean, *SD* standard deviation; ** *p* < .01; *p < .05

Pearson bivariate correlations were calculated to examine the relationships among stress, social support, self-efficacy, physical activity and QOL. These values are presented in Table 2. As shown, stress is negatively associated with social support, self-efficacy, and QOL. Consistent with the theoretical prediction, social support was positively correlated with self-efficacy, physicals activity, and QOL. Self-efficacy was positively associated with physical activity and QOL. Further, physical activity was also positively related with QOL in the present study.

### Structural equation modeling (SEM)

**Fig. 2:**
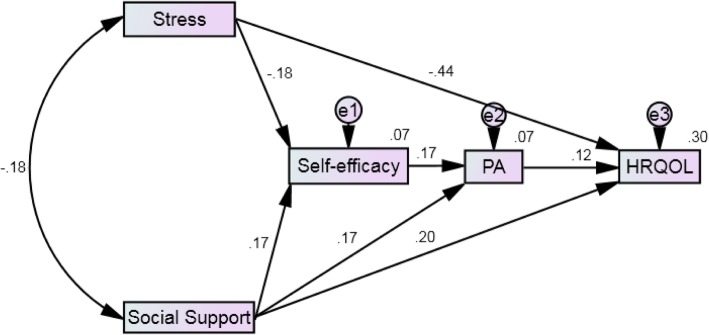
Final Model of the Variables (N = 238). *Note.* Solid lines represent significant standardized parameter estimates. Squares represent observed variables. PA = physical activity; HRQOL = health-related quality of life

SEM was used to analyze the relationships among stress, social support, self-efficacy, physical activity and QOL. Based on the goodness-of-fit statistics, the sample covariance matrix showed an acceptable fit to the hypothesized structural model (e.g., χ^2^ = 1.02; *RMSEA* = .01 [.00 .11]; *CFI* = 1.0; RFI = .96; *NFI* = .99; *GFI* = .99) [42]. Figure 2 exhibits the standardized parameter estimation of the model. All parameter estimates were statistically significant (*p* ≤ .05) with appropriate magnitude and direction. Stress and social support had direct relationships on self-efficacy (*β* = −.18 and .17, respectively). Social support had a direct relationship on physical activity (*β* = .17). Similarly, stress, social support, and physical activity had a direct relationship on QOL (*β* = −.44, .20, .12, respectively). The variance explained in the dependent variables by the model was as follows: η^2^ for self-efficacy = 7%, η^2^ for physical activity = 7%, and η^2^ for QOL = 30%. Stress has slight indirect effects on QOL, but the mediating role of self-efficacy and physical activity on QOL was supported.

## Discussion

The main purpose of this study was to investigate the psychosocial predictors of physical activity and QOL, and to examine the mediating effects of exercise self-efficacy and physical activity on the relationships between stress, social support and QOL among Shanghai working adults. The findings from this study demonstrated that stress had directly negative relationships with self-efficacy and QOL, respectively, which verified the hypothesis 1. Social support had directly positive relationships with self-efficacy, physical activity, and QOL, respectively, and it verified the hypothesis 2. Physical activity had a directly positive relationship with QOL among Shanghai working adults. Furthermore, both self-efficacy and physical activity mediated the influences of stress on QOL and social support on QOL, respectively, which verified the hypothesis 3. In addition, there was no significant difference between different age groups in terms of stress, social support, self-efficacy, physical activity and QOL.

Physical activity is an effective way to improve functional capacities and to increase QOL in older adults [15, 41]. The present study indicated that more active regular physical activity was positively related to higher levels of QOL, which is in accordance with previous studies [7–10, 45]. Furthermore, the results showed that social support was positively related to QOL, and stress was negatively related to QOL. Adults with lower levels of stress and greater social support had higher levels of QOL. These findings are supportive of prior studies [1, 15, 33, 39, 46].

The motivation and adherence of physical activity were related to many factors, such as age, vocation, gender, socioeconomic status, and physical or psychological states [11]. The findings of this study showed that physical activity had directly positive relationships with self-efficacy and social support, which were the main psychosocial predictors of physical activity. Compared with healthy people, decreased exercise tolerance, muscle dysfunction and other symptoms are main factors leading to a lower physical activity level for those with chronic disease [47, 48]. The relationship between self-efficacy and physical activity reported in this study is consistent with Mailey and McAuley [10]. Social support had a directly positive relationship with self-reported physical activity. Those with greater social support demonstrate higher levels of physical activity engagement [4, 33]. The results also indicated that social support plays an important role in facilitating physical activity, which is keeping with previous study [4].

According to the self-efficacy theory, self-efficacy is based on the hypothesis that individuals can self-regulate their own motivations and behaviors [31], which is related to the belief in one’s ability to conduct a challenging task despite barriers and adverse experiences. Self-efficacy has been shown to be one of the significant and consistent predictors of physical activity in adults [32]. In addition, there is evidence suggesting that interventions aiming at enhancing self-efficacy can effectively reduce the dropouts of adults from daily exercise [33]. Moreover, individuals feeling more efficacious about their exercise performance should be more apt to engage in self-regulation, and try to build beneficial exercise environments such as getting supports from family and friends despite inclement weather or the loss of an exercise partner [4]. The results of the present study indicated that exercise self-efficacy was positively associated with physical activity in Shanghai working adults. Therefore, intervention strategies aiming to promote self-efficacy may be beneficial in maintaining long-term physical activity. Findings from the current study showed that self-efficacy was negatively related to individual’s psychological states (e.g., stress), and positively related to social support from friends, which is in consistent with previous study [20]. Those with lower stress and greater social support had higher perceived self-efficacy. This suggests that reducing stress and increasing social support may foster positive self-efficacy beliefs in working adults.

Given that exercise self-efficacy had a significant relationship to stress, social support, physical activity, and QOL, effective strategies should be adopted to increase working adults’ exercise self-efficacy including mastery experience, vicarious experiences and self-efficacy, which in turn may increase physical activity and finally improve their QOL. Self-efficacy can be accomplished by observing others successfully perform the physical activity, and obtaining verbal persuasion from peers and workmates [31]. When people approach and complete a task successfully, they may have a higher level of self-efficacy for that task. Since social support from friends played a significant role in affecting working adults’ physical activity, self-efficacy can be improved through a powerful social support system. In this system peers inspire individuals to persist in regular physical activity.

The results of this study also showed that QOL is not only directly associated with stress, social support, and physical activity, but also indirectly related to stress and social support through the mediating effects of self-efficacy and physical activity. Exercise self-efficacy acts as a mediator for the relationships between stress and physical activity, and the relationship between social support and physical activity. Consistent with previous studies [13, 14, 36], these findings suggest that the relationship between psychosocial predictors and QOL is not a simple bivariate association, but is often indirectly related and can be better expressed as following a pathway through mediating factors (e.g., physical activity, self-efficacy). These series of relationships can be best understood from a self-efficacy framework [31].

Strengths of this study include adoption of a self-efficacy framework and contemporary statistical modeling. However, we acknowledge the limitations of this study. The first limitation involves the generalizability of our findings to other populations. The participants were conveniently sampled from Shanghai, which is the economic center of China. Thus, the findings from this study cannot be generalized to working adults in other cities because of the different levels of economic development between Shanghai and other cities. Second, all the scales used in this study were based on surveys, which may lead to participants either over or under their true levels of stress, social support, self-efficacy, physical activity, and QOL. Future studies of physical activity could incorporate pedometers or accelerometers for more accurate and objective measures of physical activity. Finally, the cross-sectional research design results in difficulty establishing cause and effect relationships among the study variables. Therefore, longitudinal studies and experimental research designs are needed to further investigate changes in stress, social support, and self-efficacy over time in Shanghai working adults, and how these changes affect their physical activity and QOL throughout their life spans.

This study incorporated a range of psychosocial variables (stress, social support, and self-efficacy), physical activity, and QOL among Shanghai working adults. Our findings suggest that the relationship between each psychosocial predictor, physical activity and QOL is not a simple bivariate association, but is often indirectly related and can be better expressed as following certain pathways through mediating factors. The findings from the present study present a strong theoretical foundation for testing the relationships between psychosocial predictors, physical activity, and QOL among Shanghai working adults. There is a tendency in adults with higher stress and less social support to reduce exercise self-efficacy, which in turn improves the probabilities to decrease their regular physical activity. These reductions, in turn, provide fewer opportunities for working adults to experience successful, efficacy-enhancing behaviors leading to further reductions in exercise self-efficacy. Our findings suggest that such declines contribute to subsequent reductions in physical activity and ultimately QOL.

## Conclusion

This study examined the psychosocial predictors of physical activity and QOL in Shanghai working adults based on the self-efficacy theory, and identified potential mediating variables among their relationships. The findings of this current study highlight an important starting point in attempts to address the relationships between psychosocial predictors, physical activity and QOL among Shanghai working adults, which is a vital aspect of physical activity and public health.

## Abbreviations

AMOS: Analysis of Moment Structures
CFI: Comparative Fit Index
GFI: Goodness of Fit Index
HRQOL: Health-related quality of life
NFI: Bentler-Bonett Non-normed Fit Index
RFI: Relative Fit Index
RMSEA: Root Mean Square Error of Approximation
